# Sustainability of Leisure Tourism Events from a Destination Social Responsibility Perspective: Do Attribution Theory Dimensions Matter?

**DOI:** 10.3390/ijerph20064847

**Published:** 2023-03-09

**Authors:** Zakya E. Y. Maki, Thowayeb H. Hassan, Mohamed Y. Helal, Mahmoud I. Saleh

**Affiliations:** 1 Mass Communication Department, College of Arts, King Faisal University, Al Ahsa 400, Saudi Arabia; 2 Social Studies Department, College of Arts, King Faisal University, Al Ahsa 400, Saudi Arabia; 3 Tourism Studies Department, Faculty of Tourism and Hotel Management, Helwan University, Cairo 12612, Egypt; 4 General Management Department, Institute of Management, Economics, and Finance, Kazan Federal University, 420008 Kazan, Russia; 5 Hotel Management Department, Faculty of Tourism and Hotel Management, Helwan University, Cairo 12612, Egypt; 6 Graduate School of Management, Saint Petersburg State University, 199004 Saint Petersburg, Russia

**Keywords:** tourist behavior, attribution theory, tourism events, corporate social responsibility, tourist personality, tourism information

## Abstract

Although Destination Sustainable Responsibility (DSR) has become a critical factor in upholding tourists’ satisfaction and positive behavioral outcomes, research on how tourists perceive different attributional dimensions (e.g., controllability, stability) about the existing information adequacy on tourists’ behavior is limited. Additionally, no study has investigated how DSR influences leisure tourists’ satisfaction across various characteristics. Therefore, the current research has the novelty of examining the effects of Destination Sustainable Responsibility (DSR) on leisure tourists’ satisfaction. The study reveals two attribution theory dimensions, controllability, and stability, as mediators and information adequacy as a moderated mediation. Additionally, the study investigates how tourists’ personalities (extroverted, conscientious, neurotic, open, and agreeable) affect their perceptions of attribution dimensions. A quantitative analysis of 464 tourists who experienced leisure activities in sustainability resorts in the Red Sea was conducted to explore these relationships. The results provide a better understanding of how DSR affects leisure tourists’ satisfaction and how different personalities influence their perceptions. Our research findings demonstrate that tourists’ perceptions of destination sustainability initiatives (DSR) are contingent upon the controllability and stability of events and that extraverted and conscientious tourists reach different attributions on DSR than those with neuroticism and openness levels and agreeableness. Additionally, it appears that information adequacy concerning the controllability of events is privileged over the event’s stability about informant amount with DSR. We explore the implications of our conclusions from both theoretical and management perspectives.

## 1. Introduction

The leisure concept in tourism studies could be experienced as emotive responses to freedom, pleasure, and feelings of control [1,2]. From a leisure service provider perspective, leisure tourists increasingly emphasize participation in active leisure activities, such as water sports activities, parties, bowling, hiking/walking, skating, bicycling, horseback riding, golfing, curling, skiing, and swimming. Adopting active leisure activities encourages leisure service providers to strategize tourists’ event experiences to maintain positive experiences [3]. According to Pergams and Zaradic [4], some tourists prefer to stay home instead of traveling and engaging in leisure activities to promote electronic entertainment. However, most individuals choose to interact physically with nature and engage in leisure activities [1]. Although destination leisure event activities have significantly increased in tourism destinations, leisure tourism literature has not investigated leisure tourists’ perception of social responsibility initiatives by leisure resorts [5]. The literature also lacks investigation of leisure tourists’ personality differences in perceiving resorts’ social responsibility initiatives concerning destination leisure events’ nature (unpredictable events).

Tourism scholars have highlighted that the big umbrella of social responsibility initiatives is the concept of corporate social responsibility (CSR) [6]. Corporate social responsibility is defined as initiatives that advocate the extension of responsibilities beyond profitability tendencies to help society and other stakeholders represent a better life for host destinations and tourists [7]. For the importance of social responsibilities on tourist attitudes, recent tourism scholars have developed the destination social responsibility concept (DSR); the DSR concept has stemmed from the (CSR) concept [8,9]. Leisure tourism resorts could engage in initiatives from different perspectives: economic (e.g., local profits and helping locals to have professional training), environmental (e.g., conservation of host destination environmental assets), and social (e.g., enhancing locals’ well-being) [10,11]. According to Su et al. [12], all these perspectives represent a critical tendency to maintain tourists’ positive attitudes toward the host destinations. Consumer behavior literature has highlighted that when service providers engage in social responsibility initiatives, individuals are likely to have positive behavioral outcomes [13,14]. Hence, the current study investigates leisure tourism resorts’ destinations’ engagement in social responsibilities’ impact on leisure tourists’ behavioral outcomes.

Importantly, although tourism leisure resort initiatives may enhance tourists’ behavioral outcomes, tourism management literature has emerged to confirm that tourists are not entirely rational but get affected by different events during holidays [15]. Thus, social initiatives may not positively influence tourists’ behavioral outcomes if events are unstable. Leisure tourists become more familiar with the nature of concrete fears because destination leisure events’ nature could be unpredictable [16]. Leisure tourists during holidays may encounter risks, and they tend to avoid them by targeting destination leisure events that could be more stable and controllable [17], leading them to not care about service providers’ initiatives toward destinations (e.g., CSR initiatives) [18]. Therefore, the current study adds additional contributions by studying the interdependence of destination leisure events’ nature of stability and controllability besides leisure resorts’ engagement in social responsibility on tourists’ behavioral outcomes. We employ attribution theory dimensions to study the events’ nature because attribution theory has three main dimensions: (locus of control—it is related to who we should assign the responsibility for various events); (stability—it is associated with the condition under which events are recurring); and (controllability—it is related to the condition under which events are under service providers’ control) [19,20]. We employ the last two dimensions to achieve the study’s second contribution. 

Notably, while investigating two attribution dimensions (controllability and stability), it is crucial to examine information adequacy as a prominent tool that affects tourists’ attribution toward events [21]. According to attribution theory, information adequacy helps individuals find better justification and interpret different events [22]. Moreover, it helps the individual avoid unexpected events [23], leading to a positive attitude [22]. Hence, the current study also examines how information adequacy affects the stability and controllability of leisure tourism events. 

Moreover, a crucial point that lacks attention to leisure tourists’ attribution in different events, is tourist leisure personality. Tourism researchers have concentrated on how different personalities have different attributions toward various events. For instance, extraverted personalities seek adventure tourism with risky activities that require effort [24,25]. In contrast, introverted personalities are likely to be shy, like a quiet environment, and prefer relaxing activities [26]. However, leisure tourism researchers lack research about how different leisure tourists’ personalities affect different behavioral outcomes. According to Leung and Law [24], tourists interact and judge various activities depending on their personalities. Hence, we contribute to such a gap by studying how different personalities lead to different attributions toward leisure resorts’ social responsibility initiatives and the destination leisure event natures. 

The present study introduces a valuable contribution to tourism and event literature discourse with several important considerations. First, the correlation between tourists’ behavioral outcomes and social responsibility initiatives is examined within the leisure tourism scope. Second, this avenue of research explores social initiatives from attribution theory dimensions, such as stability and controllability. Third, we investigate the leisure tourists’ personalities to analyze their behavior towards destination leisure events and resort initiatives which is crucial due to the influence of individual characteristics on behavioral outcomes. Fourth, the study evaluates the role of information adequacy concerning the social initiative and attribution theory within the scope of tourism and leisure. Understanding and filling the gaps in these areas is highly beneficial in providing a deeper comprehension of leisure tourism engagement in the social responsibility initiatives process and how such knowledge could bring important managerial implications in tourism resorts.

From the previous contribution aims, we can identify the following research questions and objectives: Research Questions: 1. What is the correlation between tourists’ behavioral outcomes and social responsibility initiatives within the leisure tourism scope? 2. How do social initiatives from attribution theory dimensions, such as stability and controllability, impact leisure tourists’ personalities and behavior? 3. What role does information adequacy concerning the social initiative and attribution theory have within the scope of tourism and leisure? Objectives: 1. To examine the correlation between tourists’ behavioral outcomes and social responsibility initiatives within the leisure tourism scope; 2. To evaluate the impact of social initiatives from attribution theory dimensions, such as stability and controllability, on leisure tourists’ personalities and behavior; 3. To analyze the role of information adequacy concerning the social initiative and attribution theory within tourism and leisure; 4. To identify managerial implications in tourism resorts stemming from understanding leisure tourism engagement in the social responsibility initiatives process.

The outline of this academic article follows an order of study to demonstrate an overall idea from the persuasive research of attribution theory and its dimensions. In the next section, the literature review of attribution theory and its dimensions provides foundational information on the concept. Afterward, information adequacy and tourist characteristic types are highlighted for further understanding. Following this, the method section explains the methodological techniques and results with the use of SEM and ANOVA tests. Finally, the results are discussed, and a final consideration of theoretical and managerial implications is provided to conclude the article. 

## 2. Literature Review

### 2.1. Destination Social Responsibility for Tourism Leisure Resorts

Leisure conceptually identifies tourists’ mechanism to select events compatible with their priorities and life commitments, leading to leisure being a broader intrinsic concept than just enjoying holidays [2]. Leisure travel was established after the Industrial Revolution when the number of paid vacations increased. Recently, tourists have engaged in many leisure activities by participating in outdoor activities such as fishing, hunting, camping, and visiting parks [27]. Leisure activities have two types, active and passive. Passive leisure activity mode refers to tourists’ participation in non-physical exertion during holidays, contrasted against physically active leisure (e.g., reading, watching television, or hiking) [28]. However, active leisure activity refers to bodily movement engagement to significantly boost tourists’ cardiorespiratory responses, leading to psychological and physical health benefits [29]. 

Leisure tourism benefits tourism host destinations and tourists; on the one hand, from a narrow perspective, leisure tourism services providers engage in charity initiatives to enhance host community living standards and leisure tourists’ experiences. For instance, Palmer and Dwyer [1] highlighted the significance of the ‘fitness philanthropy’ term, which describes how social responsibility by destination leisure events has evolved as ‘philanthropic solutions’ to achieve social/health concerns and display ‘good’ citizenship civic responsibility engagement. Additionally, previous leisure tourism literature has focused on charity events (e.g., sports competitions) that enhance residents’ interest in leisure service providers and their positive intentions toward tourism events. Destination leisure events also attract tourism suppliers as a charity integral fundraising mechanism that increases the awareness of social responsibility toward residents and environments in such leisure destinations [1]. 

On the other hand, from a broad perspective, social responsibility in tourism destinations has been given attention recently for its benefits to tourists and tourism service providers [9]. For tourism service providers, social responsibility is essential to conserve the destination’s sightseeing assets by achieving sustainability (Font et al., 2018). As for tourists, when tourists perceive that destination managers and government officials care about social responsibilities toward different destination aspects (e.g., economy, environment, and social), the probability of holiday success will be inevitable [30]. These advantages of social responsibilities influence tourism scholars to examine the corporate social responsibility (CSR) concept, which has been developed into the concept of destination social responsibility (DSR) [6,9,12,22]. DSR could be economical (e.g., tourism destination managers’ efforts to keep track of long-term destination economic development); DSR could also be environmental (e.g., protecting biodiversity and natural resources of the tourism destination), and DSR could be social (e.g., preserving destination heritage of values and culture) [14]. Additionally, engaging in social responsibility activities helps enhance tourists’ experiences by facilitating all destination assents to remain positive experiences [10]. 

The information collected and covered in this section lends additional credence to the idea that the proactive efforts of destination stakeholders toward destination sustainability and responsibility (DSR) lead to high tourist satisfaction levels. Tourists prefer to classify DSR activities as part of their vacation experience when destinations take actions to protect the interests of the destination [6,9,19]. Additionally, these DSRs greatly influence how tourists view destination stakeholders involved in such initiatives [10]. Tourists show pleasant feelings as part of their positive response due to maintaining a sense of duty and dedication to the preservation of the host place [6]. Tourists’ satisfaction with their vacation experience is influenced by their feelings of excellence [18]. This demonstrates that the likelihood that tourists will be satisfied with their vacation experience increases the responsibility and dedication to sustainable practices in a host site and the stakeholders within it. When destinations engage in such activities to protect destination interests, tourists tend to assess these DSR activities during holidays [6,9,19]. DSR initiatives have a crucial impact on tourists’ perceptions of destination stakeholders who engage in such initiatives [10]. In turn, tourists feel responsible and committed to preserving the host destination, leading them to enact distinctive positive responses with positive emotions [6]. Positive emotions drive tourists to have a high level of satisfaction [18]. Therefore, we can hypothesize that:

**H1.**  *“Leisure tourists’ perception about resorts’ social responsibilities positively influence their satisfaction”*.

The current satisfaction assumptions about leisure resorts’ DSR advantages on tourists lack investigation of events’ incidents that may be a barrier between leisure resorts’ DSR initiatives and leisure tourists’ satisfaction. These barriers are mostly related to tourism destination leisure events’ natures. Destination leisure events could be unpredictable in stability and controllability [16]. Therefore, it is crucial to study destination leisure events’ nature in terms of stability and controllability [21] along with leisure resorts DSR.

### 2.2. Resorts’ Leisure Tourism Events Stability and Controllability

Active leisure activities require physical exertion and active leisure tourists are likely to engage in initiatives they encounter during holidays [29]. However, in practice leisure contexts suggest that leisure tourists may experience unpredictable events over their level of skill acquisition and the physical exertion needed [5]. Leisure organizers must keep controlling and stabilizing the destination leisure events’ risks that leisure tourists may encounter during holidays [5]. Leisure tourists could perceive the different types of risks such as property risks (e.g., loss of luggage and theft), planning risks (e.g., inexperienced operator, unreliable airline, and not assured flight home), political risks (e.g., political instability, terrorism, and war/military conflict), health risks (e.g., life-threatening diseases, lack of access to healthcare, and lack of access to clean food and water), and environmental risk (e.g., landslides, and natural disasters) [16]. Thus, controlling and stabilizing destination leisure events is crucial to avoid risks, especially from the attribution theory perspective.

Attribution theory provides insights into the actions of leisure travelers. Tourists frequently use this hypothesis to explain the reasons behind their experiences of a place, such as why something excellent or unpleasant happened [31]. One of the most critical aspects of understanding visitor behavior towards CSR programs is the controllability and characteristics of the theory of attribution of stability [32]. The idea of attribution theory relies on psychological concepts that pinpoint the data individuals rely on to determine whether an event or group of events caused an event. There are other types of attribution, such as internal attributes—when an event is believed to be the result of a person’s choices and intentions—and external attributes—when an event is believed to be triggered by external forces. According to the idea of attribution, visitors are more likely to support CSR activities if they attribute their motivation to internal rather than external sources [33,34]. The controllability attribution dimension explains how travelers map controllable elements of a destination, such as tourism personnel, infrastructure, services, brands, management strategies, physical environments, social environments, legislation and regulations, and destination tourism policies, to their experiences. A tourist’s assessment of the control over his experiences at a destination depends precisely on how much control he believes he has over these elements [35,36]. They are more likely to attribute their positive experiences to variables they could control if they thought they had that power. On the other hand, if they believe they do not influence these things, they may blame them for their unpleasant experiences.

The attribution of the stability dimension shows how tourists relate to their experiences [31]. These temporal or situational aspects provide stability and continuity in their experiences and the success of a CSR activity. Settlement referral dimensions consider variables such as seasonal challenges, destination activity levels and tourist seasons, population, economic, competition, social, and cultural factors, turnover rates, location and geography, infrastructure and services, visibility and reputation within the destination, and industry trends within the goal [37]. Tourists rate the stability of a destination based explicitly on their perception of their level of stability about these parameters. They are more likely to attribute their positive experience to these characteristics related to stability if they believe the destination has a high level of stability [31]. They may blame these elements for their unpleasant experience if they believe there is a low level of stability. Understanding how tourists attribute the cause of events or behaviors and how these attributes influence their behavior is therefore essential to accurately predicting how visitors will respond to CSR activities. The controllability and stability features of attribution theory allow us, in particular, to assess the effectiveness of individual CSR and to better understand the driving forces behind visitors in predicting their behavior [34].

In the vein of tourism events, the event stability dimension reflects the perceived extent to which the events are unstable over time (temporary) or stable over time (permanent) [20]. The stability in positive events (vs. negative) stimulates individuals to believe they will receive the same experience in the future. According to Dunn et al. [38] and Swanson and Kelley [22], individuals are more likely to become satisfied with the positive, permanent cause (vs. temporary). Individuals tend to avoid risks, so they prefer stable positive events to avoid losing reliability and confidence in the suppliers’ abilities [39]. Hence, individuals prefer stable services, giving them a superior level of positive behavioral outcomes [40]. 

Whereas the event controllability dimension reflects the perceived degree to which events are under service providers’ control [38]. Several attribution studies have revealed that consumers feel disappointed when service providers cannot control service failure [38,40]. Conversely, consumers are likely to be tolerant if they feel that the service providers have little control over the failure of the perceived services [18]. Thus, when consumers feel that service providers care to avoid triggering adverse reactions toward uncontrollable negative events, they are more likely to have positive behavioral outcomes [20,41]. 

In this vein, as tourism is considered one of the industries, tourists may encounter unexpected events [18,20,42,43]. Leisure tourists attribute the tourism event to stability and controllability before their final judgments. For instance, when leisure tourists perceive that service providers stabilize and control destination leisure events’ outcomes, they are more likely to have positive behavioral outcomes.

Therefore, we hypothesize that:

**H2a.**  *“Leisure tourism events’ stability positively influences tourists’ satisfaction”*.

**H2b.**  *“Leisure tourism events’ controllability positively influences tourists’ satisfaction”*.

When tourists perceive controllable and stable positive events, they are likely to perceive service providers’ side initiatives (leisure resorts DSR initiatives) (Jackson, 2019). Whereas tourists who perceive that the positive (vs. negative) events are temporary and uncontrollable are more likely to have adverse behavioral outcomes toward service providers [44], building barriers to perceiving their initiatives (leisure resorts DSR initiatives) at the destination. Therefore, destination leisure events’ stability and controllability could bridge perceiving resorts’ leisure DSR initiatives, leading to tourist satisfaction. It could also be a barrier preventing tourists from perceiving DSR initiatives in unstable or uncontrollable events, leading to tourist dissatisfaction. Therefore, we suggest the following hypotheses:

**H3a.**  *“Leisure tourism events’ stability positively mediates the relationship between leisure tourists’ attribution toward DSR initiatives and their satisfaction”*.

**H3b.**  *“Leisure tourism events’ controllability positively mediates the relationship between leisure tourists’ attribution toward DSR initiatives and their satisfaction”*.

### 2.3. Information Adequacy as a Moderator

As pointed out earlier, events’ stability and controllability are dimensions of attribution theory and affect tourists’ attribution. According to attribution theory, information affects individuals’ cognitive processes [23]. Lack of knowledge about various events may endanger individuals that encounter risks during service encounters [15]. This perception of inadequate information about events is independently related to improper behavior when experiencing unexpected events during service encounters [45]. Hence, attributing different occasions with a lack of knowledge has a scant influence on the post-attribution behavior than attributing events with sufficient information [45]. This is because information adequacy considers one of the most significant antecedents of cognitive attribution processes [46]. Against this background, this current study examines the effects of information adequacy about tourism events as a decisive factor affecting tourism destination leisure events’ stability and controllability on tourists’ behavioral outcomes.

Thus, the study hypothesizes:

**H4.**  *“Information adequacy strengthens the relationship between events’ stability, controllability and tourists’ satisfaction”*.

Tourism researchers have concentrated on how different personalities have different behavioral outcomes when experiencing many travel events [47]. Thus, it is crucial to examine tourist personalities and apply them empirically to the leisure context.

To accurately and effectively assess how information adequacy impacts tourists’ satisfaction and loyalty, it is essential to investigate tourist traits along with attribution theory and social responsibility initiatives. Tourists’ personalities and personality differences are strongly associated with their tendency to attribute different events differently, which can significantly impact leisure tourists’ satisfaction. For example, “Extraversion tourists” are more likely to feel pleasure when attributing various events and sharing their experiences from the destination. In contrast, “Neuroticism tourists” tend to attribute different events critically. Furthermore, “Openness tourists” are more likely to evaluate their expectations for novelty critically, and “Agreeableness tourists” are more likely to attribute different possibilities to obtain positive experiences positively. Lastly, “Conscientious tourists” are more likely to attribute events to improve experiences accurately. Therefore, investigating the influence of tourist traits in combination with Attribution Theory and social responsibility measures is essential in predicting tourist behavior.

### 2.4. Tourists’ Personal Traits

Tourists’ personalities indicate their behavior toward different events [18]. There are many types of tourists’ personalities; first, “Extraversion tourists” are more likely to be assertive, adventurous, sociable, talkative, and active [24,48]. This personality type is more likely to feel pleasure while attributing different events to help improve their experiences and/or service providers’ efficiency by sharing information about their holidays with others [47]. Second, the “Neuroticism tourists” more easily have worried, unstable, sad, and temperamental moods [47,49]. This personality type is more likely to paradoxically and critically attribute different events that may affect services’ judgment and attribution processes [24,48]. Third, the “Openness tourists” are more likely to have broad interests, curiosity, and divergent novel thinking [47]. This personality type is more likely to critically attribute different events than other tourists to evaluate their expectations regarding divergent novel thinking [50]. Fourth, the “Agreeableness tourists” naturally have favorable judgments toward events [49]. This personality type is more likely to positively attribute different possibilities to acquire positive experiences than other tourists [47]. Fifth, “Conscientious tourists” are more likely to be strong-willed, purposeful, and determined [49,50]. This personality type is more likely to accurately attribute different events to improve experiences than other tourists [47]. These diverse personalities will help discover new insights into how leisure tourists behave toward various events. Therefore, besides the study model, we develop a post hoc analysis to investigate the leisure tourists’ personality behavioral outcomes toward destination leisure events and resorts’ DSR.

The current literature on leisure tourists is relatively well-established, focusing on tourists’ attitudes and behavior, decision-making processes, influences, and motivations. However, there is a gap in the literature on the breakdown of attributional dimensions of controllability and stability, their relation to information adequacy, and the resulting impact on leisure tourists’ satisfaction. This distinct gap needs to be addressed so tourism destination resort managers can have concrete information to make decisions about improving their clients’ satisfaction. Generally, attributions of controllability and stability are seen as predictors of tourists’ satisfaction and loyalty. However, there has yet to be research into how tourists may perceive the controllability and stability of destination leisure events when they encounter different levels of information adequacy. The current study seeks to fill this gap to uncover how information adequacy impacts tourists’ perceptions of controllability and stability and how such attributions impacted the overall satisfaction of travelers.

**Figure 1:**
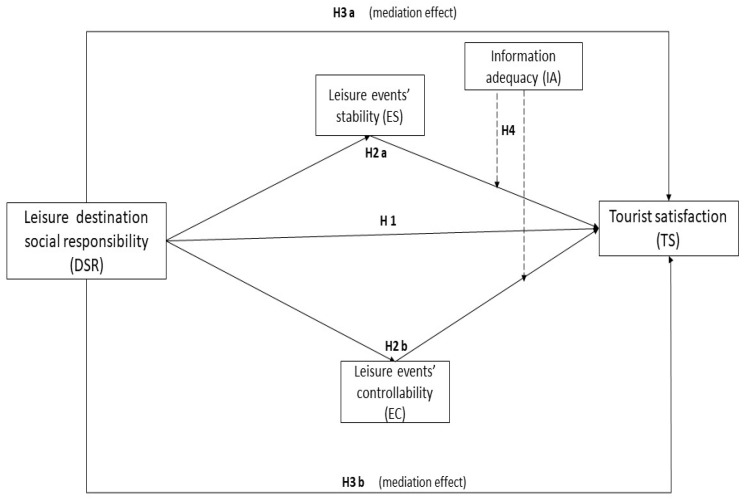
The Proposed Model.

Moreover, this study is the first to treat and study the tourists’ characteristics regarding their perception of DSR, considering attribution dimensions and information adequacy. The potential results can be performed to inform tourism resort managers of how they can adjust their methods of providing tourists with information while they are on vacation to increase their satisfaction and loyalty effectively. Furthermore, this research could also provide insight into the human experience as it relates to leisure travel, shedding light on how humans respond to different levels of control and stability when away from home and potentially providing helpful information regarding similar scenarios that might occur in everyday life, considering their other traits. As such, this research is vital to understanding and improving travelers’ satisfaction and has meaningful applications in various settings. By adopting the quantitative method, we developed a new structural equation model and post hoc analysis to address the current study’s contributions (see Figure 1).

## 3. Materials and Methods

### 3.1. Construct Measurements

The current study introduces four items as the social responsibility construct measure. It belongs to Su et al. [6], Su and Swanson [9], and Su et al. [12] literature assumptions about DSR consequences on tourist behavior when enhancing tourists’ attribution toward different events. Additionally, the study adopts three items from Jackson [18] and Fong et al. [44] to clarify both events’ stability and controllability as crucial dimensions within attribution theory to treat them as mediators between DSR and tourists’ satisfaction. As for tourist satisfaction, three items are adapted from the literature [18,51] to understand how attribution dimensions and DSR initiatives influence tourist satisfaction. Finally, for the moderator (information adequacy), three items are adapted from García-Milon et al. [23] to test how information about tourism events could strengthen the relationship between events’ stability, controllability, and tourist satisfaction.

### 3.2. Data Collection and Pre-Tests of Measurement

An online survey was conducted to examine the study model and empirically assess the reliability and validity of the developed scale. The survey of this study was conducted during the summer months of June 2022 to August 2022. A multinational travel agency specializing in Red Sea resorts conducted the survey in English. Tourists who have experienced sustainable activities and environmental campaigns in the Red Sea destinations are the primary target of this survey. We thoroughly evaluated the title, questions, and answer choices in the questionnaire by tourism experts to ensure that each item was clear, concise, and relevant to the study’s objectives. Experts reviewed the clarity of each question and the depth of options available regarding the sustainability of leisure tourism events from a destination social responsibility perspective: Do dimensions of attribution theory matter? They gave their opinions on the public perception and relevance of the questionnaire to ecotourism topics and perspectives of attribution theory. Based on their feedback, any necessary changes or modifications were made to the questionnaire. After this step, the questionnaire was tested on a representative sample, and any modifications or further modifications were made considering the test results. The criteria for selection as a survey participant include experienced tourism activities in the Red Sea area and traveling to the Red Sea. We informed tourists of the study’s aims and scope before they had the survey link. We determined the minimum sample size based on multiple criteria to prevent potential difficulties associated with the validity of statistical inference. We followed Netemeyer et al. [52] and Cattell’s [53] suggestions to ensure sufficient power for the statistical measurements to obtain an unexpected ratio. Thus, we set our target at 550 tourists, we targeted this number because of the various propositions that have been made regarding determining a suitable sample size for Structural Equation Modeling (SEM). For instance, Boomsma [54,55] proposed that a minimal adequate sample size should be at least 100 or 200, and Bentler and Chou [56] suggested a ratio of five observations or ten estimated parameters. A popular heuristic proposed by Nunnally [57] involves using ten cases per variable. Although such rules provide a basis for sample size estimations, they are not universally applicable. They can either overestimate or underestimate the number of observations required for reliable estimates for a particular SEM. Wolf et al. [58] acknowledged that specific characteristics of a certain model, namely commonality across the variables, sample size, and degree of factor determinacy, have a significant effect on the accuracy of parameter estimates and accompanying fit statistics, thereby raising doubts as to the applicability of sample size rules of thumb. After launching the survey, we collected 464 complete surveys with a response rate of 84.4%. More significantly, we drew a pre-test of our measures sample by surveying 45 tourists to ensure validity and reliability. The pre-test measures show the reliability and validity of the measurements on Cronbach Alpha and composite reliability; both are significant at 0.7, outpacing the threshold [59] with a significance level of 0.01.

## 4. Results

### 4.1. Respondents Profile

Respondents’ demographic analysis shows that females participated in the study more than males (75.4% vs. 24.6). Given the fact that Couchsurfing users are mainly from the youth generation (O’Regan and Choe, 2019), the respondents’ highest percentages were between 19 to 30 years old (52.2%). Ages between 15 to 18 years old were 35.3%, and the lowest percentages were between 31 and 40 (4.7%). Additionally, most tourists were highly educated as follows; Licentiate/Bachelor (49.6%) and Master’s degree (24.6%); this was because over 50% of participants indicated that they were students and (31.9%) were employed (Table 1).

### 4.2. Measurement Model Test

#### The Model Reliability and Validity

We performed a normality test before running the analysis and testing the hypothesis. The results revealed that both Skewness and Kurtosis values matched Kline’s [60] normality threshold. Skewness was below 0.3, and Kurtosis was below 10 for all items. Thus, the data in this study usually had a normal distribution, leading to the subsequent analyses.

The present study utilizes the structural equations modeling (SEM) technique using AMOS 25 software to test the hypothesis. According to Mikulić and Ryan [61], SEM is one of the most popular techniques for testing social science’s variable/constructs regressions. Therefore, the study performed SEM through three main steps.

First, the study employed confirmatory factor analysis (CFA) to capture the items’ loading to the primary construct, not below (0.5), to maintain accuracy [59]. Moreover, it helped to capture the loading factor to test composite reliability and average variance extracted (AVE) [62].

Second, the study checked the reliability; Table 2 presents the constructs of Cronbach’s Alpha, which ranged from 0.72 to 0.86, outpacing the recommended threshold of 0.70 [59]. Composite reliability also ranged from 0.75 to 0.85 outpacing the recommended threshold of 0.70. Hence, the results demonstrate internal consistency in measuring the research constructs.

Third, the study examined the model’s validity for both convergent and discriminant validity. As for the convergent validity, Table 2 shows that the value of average variance extracted (AVE) for all constructs is between 0.50 and 0.70, outpacing the threshold value of 0.500 [59,63]. This indicates that the average item loading, mostly above 0.70, mostly exceeds the variance extracted, ensuring convergent validity [59].

As for discriminant validity, the study compared the square root of AVE and the coefficients’ correlations between constructs of each pair [64]. Table 3 shows that the square roots of AVEs are higher than the correlations among each construct’s pairs, assuring discriminant validity according to Hair [65].

Moreover, to assess whether external values adversely influenced the ordinal standard errors in the data, the robust standard errors—also referred to as the Huber–White standard errors—were applied to estimate the statistic’s variability that is resistant to outliers. Our results show that the strong standard errors were inferior to the regular standard errors, implying that outliers were not causing any distortion of the findings.

### 4.3. Model Fit and Hypotheses Tests

Table 4 presents the adequacy of model fit; the model fit results which have been obtained from AMOS fit standards suggested by Bagozzi and Yi [59] and Hu and Bentler [66] of χ^2^/df < 5; CFI > 0.80; RMSEA < 0.08. The other indexes are also suggested to be greater than 0.90 [59]. The results in Table 4 confirm the model fit, and the model is acceptable to test the hypothesis [59,67].

**Figure 2:**
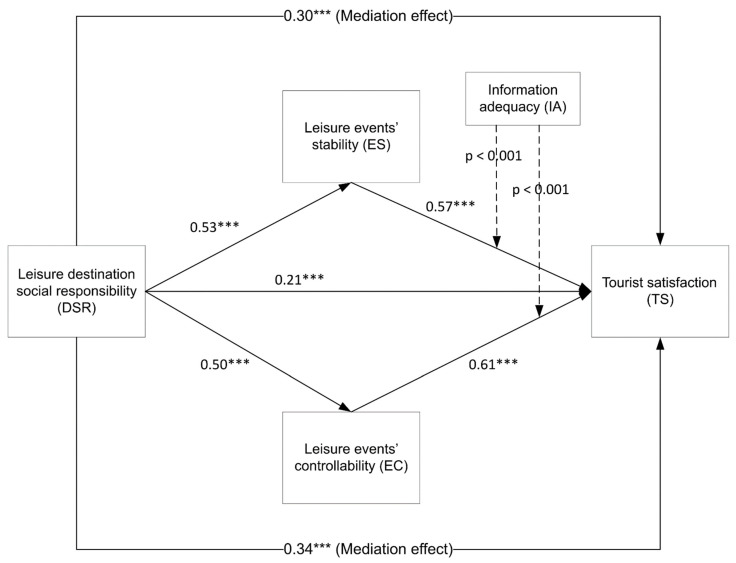
The Results of the Structural Model Test. Note: *** means significant at the < 0.001 level.

As for hypotheses tests, Table 5 and Table 6, and Figure 2 show the hypotheses tests through the direct and indirect relationship as follows: As for direct effects; The path correlations were statistically significant for H1: β destination social responsibility → tourist satisfaction = 0.21, t = 2.36, *p* <0.001; H2a: β event stability → tourist satisfaction = 0.57, t = 4.90, *p* < 0.001; H2b: β event controllability → tourist satisfaction = 0.61, t = 5.02, *p* < 0.001. As for indirect effects (mediator effects), the path correlations were statistically significant for H3a: β destination social responsibility → event stability → tourist satisfaction = 0.30, t = 4.37, *p* < 0.001, and H3b: β destination social responsibility → event controllability →tourist satisfaction = 0.34, t = 4.25, *p* < 0.001. As for total effects, the relation is statistically significant in β destination social responsibility → event stability → tourist satisfaction = 0.50, t = 6.49, *p* < 0.001, and statistically significant in β destination social responsibility → event controllability →tourist satisfaction = 0.53, t = 6.53, *p* < 0.001.

### 4.4. (Hypothesis 4) Moderated Mediation Results of Information Adequacy

**Figure 3:**
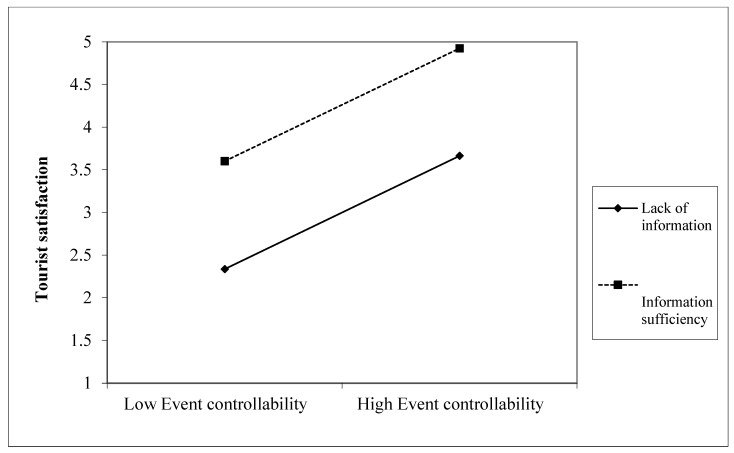
Interaction effect between information adequacy and destination leisure event controllability on tourist satisfaction.

**Figure 4:**
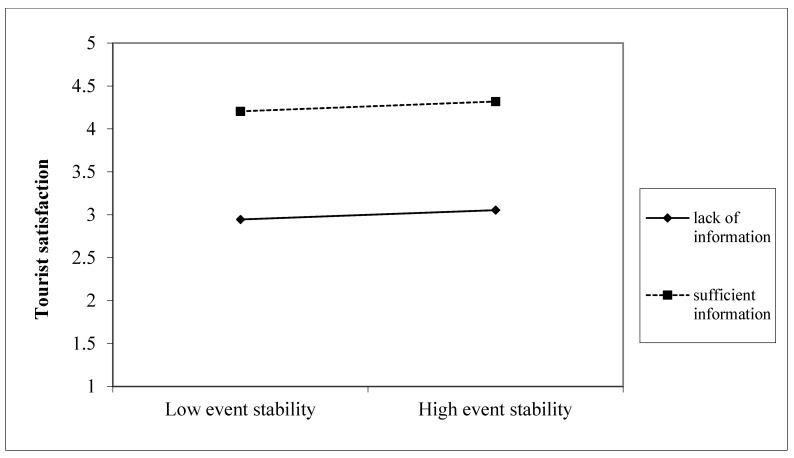
Interaction effect between information adequacy and destination leisure event stability on tourist satisfaction.

We employed PROCESS macro moderated-mediation analysis with 5000 bootstrapping procedure resamples by the 95% confidence intervals to identify how information adequacy strengthens the positivity of tourists’ satisfaction [68]. The current study examined the moderation role of information adequacy, assuming that it enhances the relationship between event stability, controllability, and tourist satisfaction. In Table 7, the results show that the interaction effect between information adequacy and event controllability on tourist satisfaction is significant (*p* < 0.001) and offers a strong tendency to have satisfaction (see Figure 3) more than destination leisure event stability. That is because the interaction effect between information adequacy and event stability shows a lower level of tourist satisfaction—compared with event controllability—at a different level of information adequacy; however, it is also significant (*p* < 0.001) (see Figure 4). This indicates that sufficient information about tourist events drives tourists to be intensely aware of destination leisure events’ controllability as a priority, then stability, leading them to intense satisfaction with service providers.

### 4.5. Leisure Tourists’ Personal Traits’ Differences (Post-Hoc Analysis)

After explaining personality synonyms to participants, we asked participants to identify their personalities, with the question “how do you consider your personality?” with five answer choices (Extroverted, Conscientious, Neurotic, Open, Agreeable). Then, we compared the mean differences to examine leisure tourists’ traits. We found that leisure tourists who engage in different leisure activities and adventures (Extraverted and Conscientious) are likely to perceive leisure resorts’ social responsibilities compared to other personalities (M Extraverted = 3.63, M Conscientious = 3.52) (Table 8). Moreover, they are more likely to encounter controllable destination leisure events than other personalities (M Extraverted = 3.51, M Conscientious = 3.58). However, tourists who don’t have strong-willed motivations to engage in different activities (just following a specific itinerary trip) or have hesitations about engaging in various leisure activities at destinations are more likely to focus on the stability of events (whether the events recur or not) than others (M Neurotic = 3.68, M Open = 3.96, M Agreeable = 3.93).

## 5. Conclusions and Implications

With the increasing number of tourists, tourism marketing scholars have opened up arguments about the importance of social responsibilities toward host destinations [19] and its impacts on tourists’ behavioral outcomes [6]. However, with the representation of leisure tourism events and spatial consumption, there is a noticeable gap in examining the social responsibility benefits of leisure tourists in a specific leisure tourism context. There is also less intention to explore the nature of destination leisure events regarding the stability and controllability concepts. Additionally, there is a wide gap in recognizing leisure tourists’ personalities regarding the events’ dynamics of the tourism experience. Therefore, the current study addresses these gaps and contributes to tourism leisure management literature as follows: 

First, we illustrate the need to study how leisure resorts’ engagement in social responsibility impacts leisure tourists’ behavioral outcomes. We have found that leisure tourists who perceive that leisure resorts engage in social initiatives (e.g., host community sustainability enhancement) are likely to have positive emotions with satisfaction. That is because social responsibility in tourist destinations has positive impacts on host destination infrastructure and superstructure improvement [9], increases resident satisfaction [19], and increases tourist engagement in destination activities [8]. Leisure tourists in nature like to engage in different sets of exercises in leisure destinations [3] and interact with the environment [2]. Therefore, when leisure tourists find resorts that interact and care about the environment and different activities in host destinations through the DSR initiatives, they are likely to get satisfied.

Second, we examined the destination leisure events regarding events’ stability and controllability through the attribution theory lens. Leisure tourism events (e.g., parties, camps, sports activities) are not stable or controllable all the time [5] because leisure tourists may encounter external or unpredictable events during holidays [1,29]. Unanticipated events are a barrier between tourists’ and service providers’ initiatives that enhance tourists’ behavioral outcomes. Therefore, we studied how destination leisure events’ stability and controllability could affect the relationship between resorts’ DSR initiatives and tourists’ behaviors. We found that leisure tourists care more about leisure activities’ controllability than stability; needless to mention that stable events are crucial, but controllability comes as a priority, according to our results. 

When leisure resorts offer diverse leisure activities that they can control, leisure tourists recognize that they are more likely to reuse these activities (stability: permanent leisure activities) [18], and they realize that the resorts can control and prevent adverse events (controllable) [42]. In this vein, leisure tourists will have satisfaction with leisure resorts. When leisure tourists are satisfied, they respond to different side activities [51]. Social responsibility activities have the most side activities that could offer a sub-optimal economic performance to tourists by providing value-added tendencies [13]. Recent literature highlighted that tourists could perceive the advantages of different destination side activities if they felt stability and controllability during their holiday [9,10]. That is why destination leisure event stability and controllability positively mediate the relationship between leisure resorts’ DSR initiatives and tourist satisfaction. 

Third, we have embedded information adequacy about destination leisure events as a moderator between tourism destination leisure events’ stability, controllability, and leisure tourist satisfaction. Consistent with recent research, information adequacy influences attribution processes [45]. Thus, information adequacy could strengthen tourists’ perceptions of destination leisure events’ stability and controllability as attribution dimensions. Our results show that information adequacy strengthens the relationship between destination leisure events’ stability, controllability, and satisfaction of leisure tourists. Tourists collect information about potential trips before traveling to destinations [69]. Tourists with sufficient information about destination events are less likely to encounter risks during holidays [70], and given that leisure tourists are likely to experience risks during holidays [16]. Therefore, with the information, leisure tourists will likely perceive stable and controllable events [71] in leisure resorts and then have strong satisfaction. 

Fourth, we found that extraverted and conscientious leisure tourists are likely affected by social responsibility initiatives and controllable events by leisure resorts. That is because the more active leisure tourists are, the more likely they are to encounter unpredictable events [29] and interact with distinct circumstances [28]. Additionally, because DSR helps prevent adverse events, they are likely to be affected by such initiatives. They also tend to find controllable events [18], because event controllability ensures positive behavioral outcomes by engaging in different holiday activities [42]. We also found that neuroticism, openness, and agreeableness focused more on destination leisure event stability. That is because these personalities are less likely to engage in different activities and make an effort during holidays; the lower engagement in destination leisure events, the less effort is made by tourists [1,5]. When tourists have low effort, they care more about securing the events than the events’ controllability [18]. Thus, leisure tourists have different behavioral outcomes depending on their personalities. 

Our study findings provide crucial theoretical contributions and managerial implications regarding the previous results. This paper is the first to contribute to tourism management literature and sustainability studies by demonstrating that tourists’ perceptions of destination sustainability initiatives (DSR) are contingent upon the controllability and stability of events and that extraverted and conscientious tourists reach different attributions on DSR than those with levels of neuroticism, openness, and agreeableness. Additionally, it appears that information adequacy concerning the controllability of events is privileged over events stability about informant amount with DSR. It is crucial to study the findings presented in this paper as they provide valuable insights into how tourists perceive DSR initiatives. By understanding how tourists perceive these initiatives, tourism managers can better tailor their strategies to meet the needs of their target audience.

Furthermore, this research provides a foundation for further research into how different personality traits affect perceptions of DSR initiatives. This could help inform future marketing campaigns and product development strategies for tourism destinations. Additionally, this research could be used to inform policymakers on how best to promote sustainable tourism practices in their regions. Overall, this paper makes an essential contribution to the tourism management literature and sustainability studies by demonstrating how different personality traits affect perceptions of DSR initiatives. This research has the potential to inform future marketing campaigns and product development strategies for tourism destinations, as well as inform policymakers on how best to promote sustainable tourism practices in their regions.

### 5.1. Theoretical Contribution

The present study contributes to leisure tourism management literature in several ways. First, it is one of the first studies to apply the corporate social responsibility concept and attribution dimensions (stability and controllability) in the leisure tourism context. However, tourism marketing and management literature have widely examined destination social responsibility effects on tourists’ behavioral outcomes [19,72]. Few studies have attempted to investigate resorts’ leisure social responsibility benefits considering destination leisure events’ nature. This research extends corporate social responsibility theory in the tourism context by examining destination leisure events’ nature (stability and controllability) as mediators between leisure resorts’ DSR and leisure tourists’ satisfaction. It adds to our understanding of how leisure tourists’ attributions about stability and controllability of destination leisure events lead to satisfaction; this contributes to the current literature on corporate social responsibility in the tourism context [8,10,19], along with leisure tourism literature.

Second, the current study is the first to highlight how different leisure tourists’ personalities have different behavioral outcomes toward social responsibilities concerning destination leisure events’ natures. We found that leisure tourists have different behavioral outcomes regarding their willingness and effort while engaging in various leisure activities. Thus, studying tourists’ personalities in the tourism leisure literature contributes to the current literature in the leisure tourism context [1,5].

Third, the study adds information adequacy as a new construct to moderate leisure tourists’ attribution processes. These results help develop a comprehensive theoretical framework and contribute to information adequacy arguments in tourism literature [23,70,71] by clarifying how leisure tourists could remain stable and controllable attributions toward leisure resorts with sufficient information about leisure tourism events. Additionally, the study confirms the service failure literature [73] by demonstrating that service stability and controllability positively influence tourist satisfaction. It also adds to service failure literature that information adequacy could strengthen the tourists’ positive attribution concerning tourism events’ stability and controllability to avoid service failure.

Moreover, this paper is the first in tourism research and destination social responsibility to address the issue of how leisure tourists’ personalities are associated with the influence of social responsibility initiatives and controllable events by leisure resorts. The findings suggest that extraverted and conscientious leisure tourists are likely to be affected by such initiatives due to their greater engagement in different holiday activities. Meanwhile, neuroticism, openness, and agreeableness focused more on the stability of the destination leisure events. This research provides valuable insight into how leisure tourists respond differently to DSR initiatives and controllable events, which might help inform the tourism industry.

### 5.2. Managerial Implications

The focus of any tourism institutional or non-institutional goals should be to promote the tourists’ and stakeholders’ well-being. The findings demonstrate that the Red Sea leisure resorts’ engagement in social responsibility initiatives will maintain tourist satisfaction. Thus, the Red Sea leisure resort managers and government officials (Ministry of Tourism) should concentrate on imposing formal and informal legislation to apply social responsibility initiatives in such resorts. For instance, the Red Sea leisure resorts’ managers may engage in Mariot’s “serve 360, doing well in every direction” strategy, and Hyatt (CSR practices) (see: [74,75]), as an example of practices that help leisure destinations to remain sustainable sites and enhance host community living standards along with leisure tourists’ satisfaction. Additionally, as local cultural exhibitions consider one of the popular initiatives companies perform to enhance host living standards, we recommend that Red Sea leisure resort managers offer local cultural exhibitions in their resorts. On the one hand, leisure tourists will indirectly engage in social responsibility initiatives. On the other hand, engaging in such activities allows tourists to perceive that destination leisure events are controllable and stable by offering such exhibitions as a leisure destinations’ side activity. 

Along with engaging in DSR initiatives, we recommend that the Red Sea resort managers maintain stable and controllable destination leisure events at the destination by providing plan B in their trip itinerary. Plan B should include different alternatives if some unpredictable events occur and may lead to unstable and uncontrollable destination leisure events (e.g., bad weather, traffic accident on trip roads, flight delays, problems in escorting process from airport to hotels, etc.). Applying such a plan (B) and informing leisure tourists about it on the destination website/or after tourists’ arrival will influence stability and controllability attribution toward events. In turn, this leads to receiving social initiatives more positively and achieving a high level of tourist satisfaction. Additionally, Jackson [18] highlighted that lack of harassment and poorly-developed infrastructure negatively influences event stability and controllability at the destination. Therefore, the Red Sea resort managers and government officials should impose stand-alone rules that eliminate harassment (verbal and nonverbal) and improve the infrastructure/superstructure (e.g., roads; it will help tourists control their holiday events).

Furthermore, according to the findings, information adequacy about the Red Sea leisure activities helps maintain stable and controllable destination leisure events, leading to satisfaction. Therefore, resort managers are recommended to provide sufficient information on their websites about the service offers and provide tourists with destination information assets (e.g., destination guide points map including all appointment points and emergency numbers). Moreover, the Red Sea leisure destination managers should develop stand-alone DSR programs, mainly as a gift to tourists, to increase tourists’ awareness about resorts’ social responsibilities activities, primarily if this DSR initiative is related directly to tourism events (e.g., getting involved in volunteer activities for volunteers’ tourism type). 

Most importantly, our findings on leisure tourists’ personalities will help the Red Sea resort managers prioritize their target tourist characters’ social responsibility and the nature of entertainment events. We recommend that Red Sea resort managers prioritize providing information about social responsibility campaigns to tourists involved in different destination leisure events. We also recommend that managers prevent any negative outcome that eliminates destination leisure events’ controllability (Plan B) for extraverted and conscientious tourists. Likewise, we recommend that the Red Sea resort managers ensure recurring events and provide a diversity of destination leisure events for neuroticism, openness, and agreeableness tourists. By implementing the above suggestions, leisure resort managers and policymakers will maintain an inclusive benefit for leisure tourists’ satisfaction.

### 5.3. Limitations and Future Research

This study has several limitations. First, the samples were drawn from leisure tourists engaged in leisure activities in the Red Sea tourism resorts. The study also used Couchsurfing’s online tourism platform, and the samples were youthly leisure tourists’. Thus, we recommend that future researchers examine other representatives from different tourism platforms (e.g., tourism bloggers groups on social media), other tourism destination types, and other destinations (e.g., business, ecotourism, etc.) by considering gender and age balance. Additionally, the current study examines only two dimensions of attribution theory as mediators (stability and controllability). Thus, future studies may consider a tourist locus of causality as a mediator to study how assigning tourism events to internal versus external causes, may lead to different levels of satisfaction and the factors that stimulate a positive tourist locus. Future studies should also include whether the events’ stability and controllability differ regarding the resorts’ social responsibility types (active CSR versus proactive CSR initiatives). These suggestions will help better understand the best ways leisure tourists perceive social responsibility initiatives to maintain their satisfaction with holistic managerial implications in such approaches. Moreover, we cannot generalize these results to the general population because the sample was mostly students. This research provides important insights into how tourists perceive DSR initiatives. However, extrapolations to the larger population should be made with caution. We suggest that future studies further expand the sample size to add validity to the results. Additionally, studies should examine the impact of personality types within different geographical contexts, as it could lead to a more nuanced understanding of the relationships between tourists’ perceptions and DSR initiatives. 

## Figures and Tables

**Table 1:** Socio-demographic characteristics of respondents.

Variable		Number	Percentage
Gender	Males	114	24.6
	Females	350	75.4
Age	15 to 18	164	35.3
	19 to 30	242	52.2
	31 to 40	22	4.7
	41 to 50	18	3.9
	51 to 60	16	3.8
Level of Education	High School	78	16.8
	Licentiate/Bachelor	230	49.6
	Master’s degree	114	24.6
	Doctorate Degree	42	9
Employment Status	Unemployed	4	0.9
	Student	234	50.4
	Self Employed	54	11.6
	Employed full time	148	31.9
	Employed part time	24	5.2

**Table 2:** Results of measurement model.

Construct	Items	Mean	SD	CR	AVE	CR
Events’ stability (ES)	It’s very likely that my holiday events will occur frequently.	3.44	1.055	0.756	0.51	0.78
	I think the same holiday events will occur again.	3.30	1.086			
	My holiday events were stable.	3.40	1.073			
Events’ controllability (EC)	My holiday experiences’ events were controllable.	3.42	0.999	0.872	0.53	0.72
	Nobody in the destination could stop my holiday events’ from happening.	3.13	1.127			
	My holiday outcome is not limited to specific situations.	3.50	1.053			
Destination social responsibility perception (DSR)	I have positive attribution toward the destination because it engages in charities activities.	3.31	1.209	0.821	0.60	0.86
	Destination engagement in social responsibilities initiatives enhances my judgments toward the destination.	3.26	1.134			
	I have positive interpretations toward holiday events in destinations which engage in social responsibilities.	3.38	1.182			
	Destination’s social responsibilities enhance my positive attribution toward the destination.	3.37	1.217			
Tourist satisfaction (TS)	I am satisfied with my holiday events.	3.97	1.015	0.879	0.71	0.80
	I have enjoyed my holiday events.	3.77	1.222			
	I am satisfied with my journey	4.00	1.071			
Information adequacy (IA)	Information about my holiday events is sufficient.	3.57	1.054	0.880	0.70	0.79
	Information about my holiday events is updated regularly	3.39	1.030			
	Information about my holiday events is complete and detailed	3.33	1.063			

AVE: Average variance extracted; CA: Cronbach Alpha; CR: Composite Reliability.

**Table 3:** Correlation coefficients and average variance extracted.

Coefficients	DSR	ES	EC	TS	IA
DSR	**0.78**				
ES	0.526 **	**0.713**			
EC	0.486 **	0.602 **	**0.722**		
TS	0.467 **	0.508 **	0.509 **	**0.84**	
IA	0.489 **	0.573 **	0.531 **	0.581 **	**0.83**

Notes: square root of average variance extracted (AVE) is shown on the diagonal of the matrix (bold); inter-construct correlations are indicated off the diagonal; ** correlation is significant at the 0.01 level (2-tailed).

**Table 4:** Model fit indicators and associated evaluation criteria.

Fit Index	Criteria
χ^2^/df = 3.581	<5.00
RMSEA = 0.08	<0.09
IFI = 0.903	>0.900
TLI = 0.900	>0.900
CFI = 0.901	>0.900

**Table 5:** Structural model test results and hypothesis test outcomes.

Hypothesis	Constructs’ Correlations	β	SE	T-Value	*p*-Value	Hypothesis Test Outcome
H1	DSR → TS	0.21	0.089	2.36	0.018 *	Supported
H2a	ES → TS	0.57	0.11	4.90	0.000 ***	Supported
H2b	EC → TS	0.61	0.122	5.02	0.000 ***	Supported
H3a	DSR → TS (with ES presence)	0.30	0.06	4.37	0.000 ***	Supported
H3b	DSR → TS (with EC presence)	0.34	0.08	4.25	0.000 ***	Supported

Note: * means significant at the 0.05 level; and *** means significant at the 0.001 level.

**Table 6:** Direct, indirect and total effects.

Effect	Path	Standard Path Coefficient	Std. Err.	T-Value	*p*-Value	Confidence Interval
Direct effect	DSR > ES	0.53	0.077	6.82	0.000 ***	0.37 to 0.68
	DSR > TS	0.21	0.089	2.36	0.018 *	0.03 to 0.38
	ES > TS	0.57	0.11	4.90	0.000 ***	0.34 to 0.80
	DSR > EC	0.50	0.077	6.49	0.000 ***	0.35 to 0.65
	EC > TS	0.61	0.122	5.02	0.000 ***	0.37 to 0.85
Indirect effect	DSR > ES > TS	0.30	0.06	4.37	0.000 ***	0.16 to 0.44
	DSR > EC > TS	0.34	0.08	4.25	0.000 ***	0.16 to 0.45
Total effect	DSR > ES	0.53	0.077	6.82	0.000 ***	0.37 to 0.68
	ES > TS	0.57	0.11	4.90	0.000 ***	0.34 to 0.80
	DSR > TS (through the ES)	0.51	0.80	6.47	0.000 ***	0.36 to 0.67
	DSR > EC	0.50	0.077	6.49	0.000 ***	0.35 to 0.65
	DSR > TS (through the EC)	0.53	0.081	6.53	0.000 ***	0.37 to 0.69

Note: * means significant at the 0.05 level; and *** means significant at the 0.001 level.

**Table 7:** Moderated mediation results: Conditional indirect effect of leisure destination social responsibility on tourists’ satisfaction (via destination leisure events’ stability and controllability) at different levels of information adequacy.

Indirect Effect of	Values of Moderators	Indirect Effect	SE	R-sq	*p*-Value
DSR on TS via EC at different level of information adequacy	−1 SD	0.1320	0.0366	0.44	0.000 ***
	M	0.0874	0.0245		
	+1 SD	0.0728	0.0178		
DSR on TS via ES at different level of information adequacy	−1 SD	0.1130	0.0342	0.40	0.000 ***
	M	0.0657	0.0229		
	+1 SD	0.0384	0.0226		

Note: t statistics in parentheses/*** *p* < 0.001.

**Table 8:** Leisure tourists’ personality differences regarding DSR and events’ nature.

	How Do You Consider Your Personality?				
	Extraverted (Adventurous)	Neurotic (Temperamental Moods)	Open (Curiosity)	Agreeable (Positive Judgments)	Conscientious (Purposeful, Strong-Willed)
	Mean	Mean	Mean	Mean	Mean
DSR	3.63	3.12	3.27	3.20	3.52
TS	3.59	3.68	3.96	3.93	3.68
EC	3.51	3.29	3.24	3.40	3.58

## Data Availability

Data are available on request due to privacy/ethical restrictions.
